# A brain functional network feature extraction method based on directed transfer function and graph theory for MI-BCI decoding tasks

**DOI:** 10.3389/fnins.2024.1306283

**Published:** 2024-03-21

**Authors:** Pengfei Ma, Chaoyi Dong, Ruijing Lin, Huanzi Liu, Dongyang Lei, Xiaoyan Chen, Huan Liu

**Affiliations:** ^1^College of Electric Power, Inner Mongolia University of Technology, Hohhot, China; ^2^Intelligent Energy Technology and Equipment Engineering Research Centre of Colleges and Universities in Inner Mongolia Autonomous Region, Hohhot, Inner Mongolia, China; ^3^College of Computer and Software Engineering, Dalian Neusoft University of Information, Dalian, China; ^4^Engineering Research Center of Large Energy Storage Technology, Ministry of Education, Hohhot, Inner Mongolia, China

**Keywords:** brain–computer interface, motor imagery, directed transfer function, graph, brain network

## Abstract

**Background:**

The development of Brain-Computer Interface (BCI) technology has brought tremendous potential to various fields. In recent years, prominent research has focused on enhancing the accuracy of BCI decoding algorithms by effectively utilizing meaningful features extracted from electroencephalographic (EEG) signals.

**Objective:**

This paper proposes a method for extracting brain functional network features based on directed transfer function (DTF) and graph theory. The method incorporates the extracted brain network features with common spatial pattern (CSP) to enhance the performance of motor imagery (MI) classification task.

**Methods:**

The signals from each electrode of the EEG, utilizing a total of 32 channels, are used as input signals for the network nodes. In this study, 26 healthy participants were recruited to provide EEG data. The brain functional network is constructed in Alpha and Beta bands using the DTF method. The node degree (ND), clustering coefficient (CC), and global efficiency (GE) of the brain functional network are obtained using graph theory. The DTF network features and graph theory are combined with the traditional signal processing method, the CSP algorithm. The redundant network features are filtered out using the Lasso method, and finally, the fused features are classified using a support vector machine (SVM), culminating in a novel approach we have termed CDGL.

**Results:**

For Beta frequency band, with 8 electrodes, the proposed CDGL method achieved an accuracy of 89.13%, a sensitivity of 90.15%, and a specificity of 88.10%, which are 14.10, 16.69, and 11.50% percentage higher than the traditional CSP method (75.03, 73.46, and 76.60%), respectively. Furthermore, the results obtained with 8 channels were superior to those with 4 channels (82.31, 83.35, and 81.74%), and the result for the Beta frequency band were better than those for the Alpha frequency band (87.42, 87.48, and 87.36%). Similar results were also obtained on two public datasets, where the CDGL algorithm’s performance was found to be optimal.

**Conclusion:**

The feature fusion of DTF network and graph theory features enhanced CSP algorithm’s performance in MI task classification. Increasing the number of channels allows for more EEG signal feature information, enhancing the model’s sensitivity and discriminative ability toward specific activities in brain regions. It should be noted that the functional brain network features in the Beta band exhibit superior performance improvement for the algorithm compared to those in the Alpha band.

## 1 Introduction

Brain–computer interface (BCI) is a technology that directly connects the human brain to external devices, and it has gained significant attention in the fields of neuroscience and engineering (Värbu et al., 2022). BCI technology provides a novel means of communication and operation for individuals facing challenges in motor function, neurological impairments, or other physical limitations, thus enabling them to overcome difficulties in normal communication and interaction (Cheng et al., 2020; Chen et al., 2022; Davis and Meschede-Krasa, 2022; Hu et al., 2023; Wang et al., 2023). The motor imagery (MI) paradigm holds significant potential in the field of BCI, particularly in rehabilitation medicine and assisted living technology, owing to its high feasibility and adaptability (Pichiorri et al., 2015). In recent years, research on MI paradigms has been focused in two directions. One direction aims to improve the decoding algorithm, enhancing the accuracy of MI categorization tasks (Vallabhaneni et al., 2021). The other direction involves constructing a brain network model to investigate the associations and patterns of information transfer between different brain regions (Yu et al., 2022).

Despite the advancements in BCI technology for recognizing and decoding brain signals, there are still limitations in terms of accuracy and reliability. The decoding process of brain signals is prone to errors and uncertainties, resulting in less stable and reliable performance of BCI systems. While traditional algorithms like discrete wavelet transform (DWT) (Ji et al., 2019) and common spatial patterns (CSP) (Blankertz et al., 2008) are simple and convenient, they do not yield satisfactory classification results (Amin et al., 2015; Jin et al., 2021). The Filter Bank Common Spatial Pattern (FBCSP) algorithm, which combines band filtering and CSP analysis, aims to enhance the accuracy of MI recognition. However, individual differences and noise significantly impair its effectiveness. To address this problem, Mammone et al. (2023) proposed AutoEncoder-Filter Bank Common Spatial Patterns (AE-FBCSP), incorporating an autoencoder into the FBCSP algorithm, and Park et al. (2018) introduced regularization in the Filter Bank Regularized Common Spatial Pattern (FBRCSP), both of which greatly improved the classification accuracy of FBCSP. However, these approaches primarily focus on the spatial domain features of EEG signals with a single attribute and do not consider the transmission mode of brain information during MI. Deep learning, as a powerful machine learning method, has garnered significant interest and research in the field of BCI (Amin et al., 2019; Dai et al., 2020). Convolutional neural networks (CNNs) have shown great potential in capturing information in BCI (Simões et al., 2020; Borra et al., 2022; An et al., 2023). However, the performance of CNNs relies not only on the choice of convolutional kernels (Song et al., 2021) but also on the number of convolutional layers. On the other hand, recurrent neural networks (RNNs) process EEG time-series information more effectively and can be successful in classifying MI tasks (Bore et al., 2021). Lawhern et al. (2018) proposed a lightweight deep learning approach which is called EEGNet for the task classifications of EEG-based BCIs. EEGNet exhibited an exceptional generalization ability for classifying both within-subject and cross-subject tasks, even when faced with limited training data. Across various tested paradigms, such as P300 Visual Evoked Potentials, Error-Related Negativity (ERN), Movement-Related Cortical Potentials (MRCP), and Sensory Motor Rhythms (SMR), the classification accuracies of the EEGNet algorithm have consistently been superior to those of many benchmark algorithms (Lawhern et al., 2018). While deep learning has achieved remarkable results in BCI, it also faces common disadvantages, such as high data volume requirements and challenges in obtaining physiological interpretations. Brain network research methods offer a high degree of physiological interpretability. These methods view the brain as a complex network structure, where brain regions or electrodes are considered nodes, and the connections between them indicate functional or structural relationships. By applying concepts and methods from graph theory (de Vico et al., 2014) and network science, researchers can uncover the topology of brain networks, information transfer properties, and interactions between brain regions (Rodrigues et al., 2019). Most studies on functional brain networks have focused on functional connectivity metrics. Zhang et al. (2016) constructed brain networks using Pearson correlation coefficients and observed significant differences in small-world network metrics during different MI periods. Gong et al. (2017) proposed a brain functional network modeling method based on time-frequency Cross Mutual Information (CMI) and found significant differences in small-world network metrics across different tasks. Additionally, they discovered significant differences in brain response levels, reaction times, and activation targets under different tasks. Wang et al. (2022) used a phase-locked-value approach to construct functional brain networks, providing a better functional connectivity perspective for neurofeedback training. In the MI paradigm, directed causal connectivity provides insights into the causal interactions between nodes, making it more adept at uncovering hidden and overlooked connectivity compared to functional connectivity. Varsehi and Firoozabadi (2021) utilized Granger causality analysis (GC) to choose 8 channels from EEG signals, leading to enhanced model classification accuracy, specificity, and sensitivity. However, GC analysis is less suitable for non-linear signals despite its effectiveness in capturing the dynamics and temporal order of causality in EEG signals. Li and Zhang (2022) constructed a brain functional network for MI data classification using continuous wavelet transform (CWT) and symbolic transfer entropy (TE). However, it should be noted that TE is dependent on data distribution and has a high computational time complexity (Li and Zhang, 2022). In the field of BCI, directed transfer function (DTF) outperforms other effective connectivity metrics due to its ability to capture frequency-specific causality, high temporal resolution, and model simplicity. Therefore, utilizing DTF to construct brain functional networks is advantageous. Ma et al. (2022) enhanced the classification accuracy of a MI task by incorporating DTF features into an Auto-Regressive (AR) mode. However, their study did not investigate the impact of different frequency bands on classification performance. Awais et al. (2021) combined DTF with a probabilistic neural network (PNN), achieving a classification accuracy of 82.81%, thereby validating the activation of multiple brain regions during MI tasks. However, their study lacked an exploration of graph theoretic features and the number of channels.

Traditional electroencephalogram (EEG) signal processing methods, such as DWT and CSP, are limited in obtaining a satisfying classification accuracy. FBCSP, as an improved version of CSP, yields an increased accuracy but still focuses primarily on EEG’s spatial characteristics, overlooking the brain’s intricate multidimensional dynamical information. Deep learning techniques, despite significantly enhancing classification performance, however, depend heavily on large datasets and struggle with physiological interpretation. Furthermore, some studies have utilized TE to measure brain network connectivity, forming TE-based functional brain networks. However, TE’s computational demand is high, especially with large datasets, presenting a significant challenge for the computational capability of devices. Addressing these aforementioned issues, the proposed fusion method combines graph theory features with DTF features to further improve classification accuracy. The fusion method, call CSP+DTF+Graph theory feature+Lasso (CDGL) method, combined DTF’s capability to detect frequency-specific causal links and graph theory’s potential for in-depth physiological analysis of EEG signals, aiming to enhance classification precision in BCI applications and also offering a novel insight for graphic characteristics of the MI-BCI tasks. Therefore, the objective of this study is to propose and validate a brain functional network feature extraction method based on DTF and graph theory. The proposed CDGL incorporating DTF network features and graph theory features together achieves the highest classification accuracy among the other feature fusion methods. Furthermore, the study aims to assess the effectiveness of this method in classifying MI tasks with different frequency bands and channels. The research presented in this paper aims to investigate the influence of brain network features on decoding algorithms. The specific objectives are as follows: (1) test the ability of CSP, DTF and graph theory features to classify MI-EEG data (left vs. right hand MI), (2) test the ability of combination of features to classify MI-EEG data (left vs. right hand MI), including the novel method proposed in the study, on EEG data collected from 26 healthy participants and on public EEG dataset. In each comparison the impact of the channel numbers and the frequency band (alpha and beta) was investigated.

The remainder of this paper is organized as follows: Section 2 presents the classification algorithms of CSP, DTF and graph theory, the feature classification of Lasso algorithm, and the acquisition and processing method of EEG data. Section 3 shows the comparison experimental results with different feature incorporation and experimental setup. In Section 4, a thorough analysis and discussion of the results is provided. Finally, Section 5 presents the conclusions according to the aforementioned three objectives of this research.

## 2 Methods

### 2.1 Feature extraction methods

#### 2.1.1 CSP

Common spatial pattern is a commonly employed feature extraction method in the classification of MI EEG signals (Sun et al., 2022). Its fundamental concept involves projecting the data sequence onto a specific surface through the computation of a set of spatial filters. These filters aim to maximize the variance of the projections for the two categories on that surface, thereby accentuating the most distinctive features of each category. The CSP method is highly effective in extracting EEG signal features that exhibit exceptional discriminative capabilities among different categories, thereby offering robust performance for classification tasks.

The two types of EEG signal time series data, namely $X_{1}$ and $X_{2}$ , were normalized. Subsequently, the covariance matrices of the normalized data were computed using Eq. 2.1.


(2.1) $$R_{1}=\frac{X_{1}X_{1}^{T}}{\mathrm{trace}\left(X_{1}X_{1}^{T}\right)},R_{2}=\frac{X_{2}X_{2}^{T}}{\mathrm{trace}\left(X_{2}X_{2}^{T}\right)}\text{,}$$

where $X^{T}$ denotes the transpose of *X* and $\mathrm{trace}\left(X\right)$ is the trace of the matrix.

For each series of data, its corresponding covariance matrix was calculated. Subsequently, the covariance matrices of the two series of data were separately averaged and then added together to obtain the mixed covariance matrix. This calculation is demonstrated in Eq. 2.2.


(2.2) $$R={\bar{R}}_{1}+{\bar{R}}_{2}\text{,}$$

The resulting mixed covariance matrix was subjected to an eigen-decomposition, as demonstrated in Eq. 2.3.


(2.3) $$R=U\lambda U^{T}\text{,}$$

where *U* represents the eigenvector matrix of the mixed covariance matrix *R*, and λ represents the diagonal array of eigenvalues.

Next, the whitening matrix is computed from the eigenvector matrix and the diagonal array of eigenvalues, as demonstrated in Eq. 2.4.


(2.4) $$P=\sqrt{\lambda^{-1}}U^{T}\text{,}$$

A whitening transformation is performed on the two types of mean covariance matrices, denoted as $R_{1}$ and $R_{2}$ . The whitening matrices, denoted as $S_{1}$ and $S_{2}$ , are computed using Eq. 2.5.


(2.5) $$S_{1}=P{\bar{R}}_{1}P^{T},\;S_{2}=P{\bar{R}}_{2}P^{T}\text{,}$$

$S_{1}$ and $S_{2}$ are decomposed in Eq. 2.6.


(2.6) $$S_{1}=B_{1}\lambda_{1}B_{1}^{T},\;S_{2}=B_{2}\lambda_{2}B_{2}^{T}\text{,}$$

where the eigenvectors of the $S_{1}$ and $S_{2}$ matrices are the same and the diagonal array $\lambda_{1}$ and $\lambda_{2}$ consisting of the two types of eigenvalues sums to a unit array, there is the expression of Eq. 2.7.


(2.7) $$B_{1}=B_{2}=B,\;\lambda_{1}+\lambda_{2}=I\text{,}$$

When the eigenvalue of matrix $S_{1}$ is the largest and the eigenvalue of matrix $S_{2}$ is the smallest, the two types of signals can be classified using the matrix *B*. This classification enables the derivation of the projection matrix *W*, which serves as the spatial filter. The formula is shown in Eq. 2.8.


(2.8) $$W=B^{T}P\text{,}$$

The feature matrix *Z*, obtained by applying the spatial filter *W* to the two types of data, is calculated using Eq. 2.9.


(2.9) $$Z_{1}=WX_{1},Z_{2}=WX_{2}\text{,}$$

The feature matrix *Z* is logarithmically computed for variance, and the resulting values are used as a new feature denoted as *f*. The calculation process is shown in Eq. 2.10.


(2.10) $$f=\log\left(\frac{\mathrm{var}\left(Z\right)}{\sum \mathrm{var}\left(Z\right)}\right)\text{,}$$

The classification of the two types of signals can be achieved by inputting the feature vector *f* into the classifier. For a detailed mathematical discussion, see reference Koles et al. (1990).

#### 2.1.2 Dtf

The DTF method, proposed by Kamiński et al. (2001) is a universal multivariate approach for computing the directed connections between any pair of signals within a multidimensional dataset. DTF is developed based on GC theory, which has stronger robustness and directionality compared with GC analysis. The DTF algorithm is able to analyze signals in different frequency ranges, thus revealing the interaction of brain regions in different frequency bands, which is important for the study of brain activity and functional connectivity patterns in specific frequency bands.

The acquired multichannel EEG signal is denoted as *X*. Subsequently, the multivariate autoregressive model (MVAR) (Shibata et al., 2004) is used to fit the multichannel EEG data. This fitting process results in Eq. 2.11.


(2.11) $$\overset{p}{\underset{k=0}{\sum }}\Lambda \left(k\right)X\left(t-k\right)=E\left(t\right)\text{,}$$

Here, the elements in the N*N matrix $\Lambda \left(k\right)$ represent the parameters of the MVAR model, where *N* is the number of channels. The vector *E(t)* represents the multivariate zero-mean white noise. The parameter *p* denotes the order of the MVAR model, which influences the fitting performance. To accommodate subsequent computational needs, Eq. 2.12. is transformed into the frequency domain.


(2.12) $$X\left(f\right)=\Lambda^{-1}\left(f\right)E\left(f\right)=H\left(f\right)E\left(f\right)\text{,}$$

Among them, detailed information is shown in Eq. 2.13.


(2.13) $$\Lambda \left(f\right)=\overset{p}{\underset{k=0}{\sum }}\Lambda \left(k\right)e^{-j2\pi f\Delta tk}\text{,}$$

$H\left(f\right)$ is the system transfer matrix. The value of element $H_{ij}$ in $H\left(f\right)$ describes the strength of the connection between two leads with *j* as input and *i* as output (Kamiński et al., 2001). The DTF matrix can be constructed as follows (Kaminski and Blinowska, 1991). The DTF matrix can be constructed in Eq. 2.14.


(2.14) $$\theta_{ij}^{2}\left(f\right)={\left|H_{ij}\left(f\right)\right|}^{2}\text{,}$$

To alleviate the impact of singular sample data, the DTF matrix is normalized (He et al., 2011). The feature matrix is then obtained using Eq. 2.15.


(2.15) $$\gamma_{ij}^{2}\left(f\right)={\left|H_{ij}\left(f\right)\right|}^{2}/\overset{N}{\underset{m=1}{\sum }}{\left|H_{im}\left(f\right)\right|}^{2}\text{,}$$

where $\gamma_{ij}$ represents the information inflow ratio from node *j* to node *i*, with a value ranging between 0 and 1. $\gamma_{ij}$ value closer to 1 indicates that a larger proportion of information in node *i* originates from node *j*. Conversely, a value closer to 0 suggests that there is less information flow from node *j* to node *i*.

**Figure 1:**
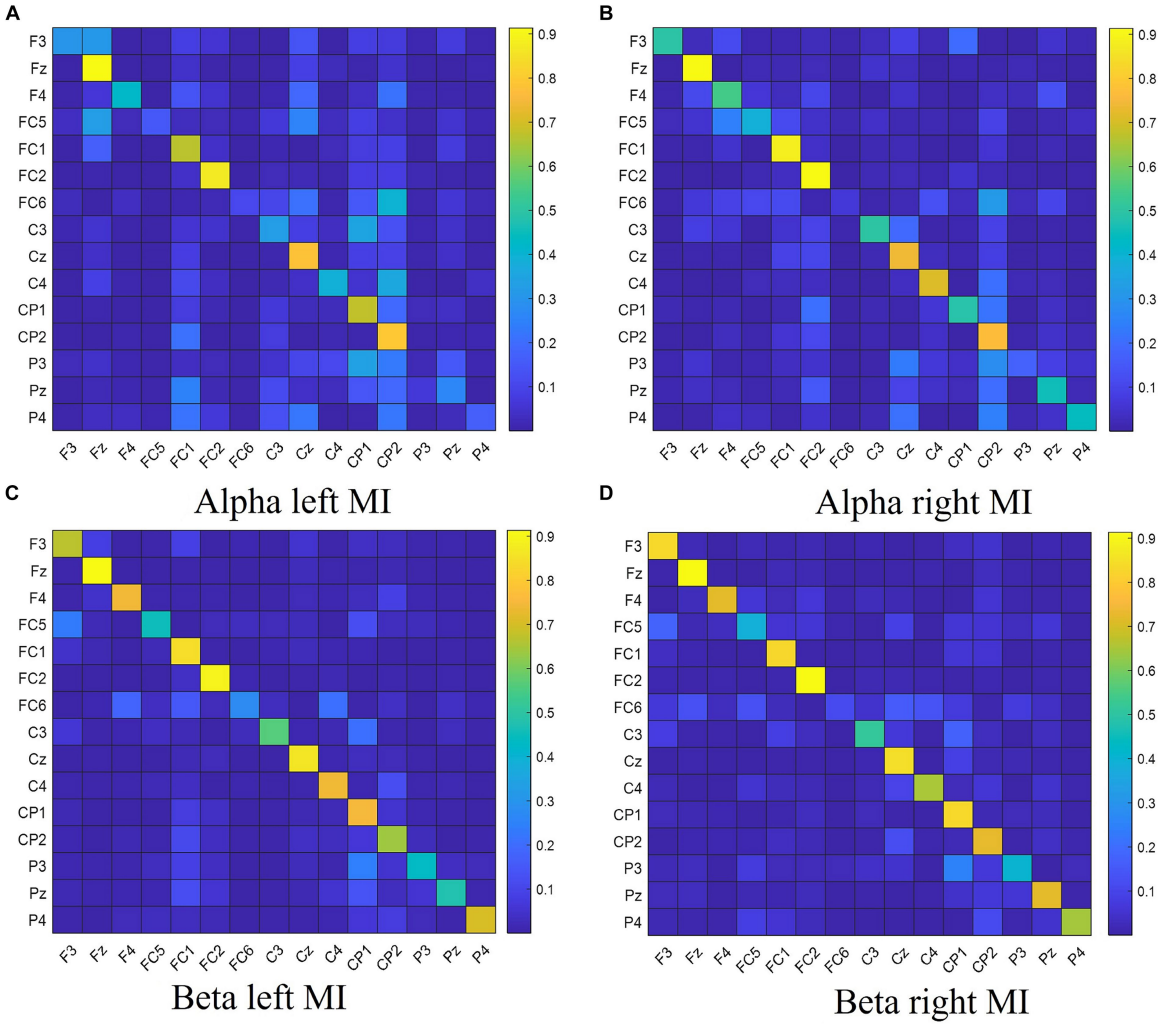
The DTF coefficient matrix of left hand and right hand MI tasks for different frequency bands. Panel **(A)** shows the DTF coefficient matrix for left hand MI in the Alpha frequency band. Panel **(B)** shows the DTF coefficient matrix for right hand MI in the Alpha frequency band. Panel **(C)** shows the DTF coefficient matrix for left hand MI in the Beta frequency band. Panel **(D)** shows the DTF coefficient matrix for right hand MI in the Beta frequency band.

The normalized DTF matrix is vectorized and utilized as feature vectors in the classifier for the purpose of classifying the MI tasks. The coefficient matrix of the 15-channel DTF network features is depicted in Figure 1.

#### 2.1.3 Graph theory method

The human brain, consisting of hundreds of millions of interconnected nerve cells, is widely regarded as one of the most complex systems in nature. This intricate neural network exhibits highly structured and functional characteristics. Through the application of graph theory, which is widely employed for the structural analysis of complex brain connectomes, we can uncover specific organizational patterns between brain structure and function. This approach provides a powerful tool to enhance our understanding of the structural connectivity networks within the brain.

Functional brain networks based on graph theory encompass crucial network features that quantify network performance. Binarizing the effective connectivity matrix, however, can lead to the loss of significant network information. In this study, the method described in Filho et al. (2018) is employed to compute characteristic parameters of the weighted network. The DTF coefficient matrix is utilized as a weighted directed graph to facilitate graph theory analysis. The graph theory metrics used in this article are as follows (Bullmore and Bassett, 2011):

(1) The calculation of ND is shown in Eq. 2.16.


(2.16) $$S_{i}=\underset{j}{\sum }w_{ij}\text{,}$$

where $w_{ij}$ is the connectivity between node *i* and node *j*, and $S_{i}$ is the node strength, which is calculated by summing up the individual weights connected to that node. The greater the node strength, the stronger the connectivity between that node and other nodes.

(2) The calculation of CC is shown in Eq. 2.17.


(2.17) $$C_{i}=\frac{\sum_{j}\sum_{k}w_{ij}w_{jk}w_{ki}}{{\left(\sum_{j}w_{ij}\right)}^{2}-\sum_{j}w_{ij}^{2}}\text{,}$$

The clustering coefficient is a metric used in graph theory to measure the degree of node aggregation within a network. It quantifies the extent to which neighboring nodes of a given node are connected to each other, thus indicating the presence of community structures in the network. A higher clustering coefficient indicates a more interconnected network.

(3) The calculation of GE is shown in Eq. 2.18.


(2.18) $$G=\frac{1}{n\left(n-1\right)}\underset{i\neq j}{\sum }\frac{1}{d_{ij}}\text{,}$$

where *n* is the number of nodes, $d_{ij}$ is the shortest path length between node *i* and node *j*. Global efficiency is a metric used to quantify the effectiveness of information dissemination in a network. It provides a measure of how efficiently information is transferred and spread across the network. A higher global efficiency indicates a more efficient and rapid dissemination of information within the network.

### 2.2 Feature selection and classification methods

#### 2.2.1 Lasso

Feature selection is a crucial aspect in the field of BCI. Its objective is to identify the most relevant and discriminative features from EEG signals, facilitating accurate classification and control of EEG signals (Lin et al., 2022). The Lasso algorithm is employed to reduce the dimensionality of the original feature space by selecting and compressing the variables (Zhang et al., 2021). The basic concept of the Lasso algorithm involves imposing a constraint on the sum of absolute values of regression coefficients, ensuring it remains below a specified threshold during the construction of a linear regression model. By applying this constraint, the Lasso algorithm effectively compresses regression coefficients with smaller absolute values to zero, thereby achieving feature sparsity and interpretability. The cost function associated with the Lasso algorithm is Zhang et al. (2021). The formula is shown in Eq. 2.19:


(2.19) $$J\left(\beta \right)=\frac{1}{2}\overset{m}{\underset{i=1}{\sum }}{\left({\hat{y}}_{i}-y_{i}\right)}^{2}+\lambda \overset{n}{\underset{j=1}{\sum }}\left|\beta_{j}\right|\text{,}$$

In the context of the Lasso algorithm, the variables are defined as follows: *m* represents the number of training samples, *n* represents the dimensionality of the original spatial features. Additionally, the cost function includes two important components: λ , which represents the weight of the penalty term and controls the dimensionality of feature selection and compression, and β , which denotes the parameter in the regression model.

#### 2.2.2 SVM

Support vector machine (SVM) is a robust machine learning algorithm that has gained significant popularity in the field of BCI in recent years (Wang et al., 2021). SVM effectively performs classification task by identifying the optimal hyperplane that separates samples belonging to different classes. It exhibits strong generalization ability and can handle high-dimensional data effectively. The underlying model of SVM is Jia et al. (2019). The formula is shown in Eq. 2.20:


(2.20) $$f\left(x\right)=\mathrm{sgn}\left[\overset{L}{\underset{i=1}{\sum }}a_{i}y_{i}k\left(x_{i}\cdot x\right)+b\right]\text{,}$$

where *sgn* is the sign function, $k\left(x_{i}\cdot x\right)$ is the kernel function, and $a_{i}$ and *b* are the parameters that determine the optimal classification plane. The kernel function takes the RBF kernel function.

**Figure 2:**
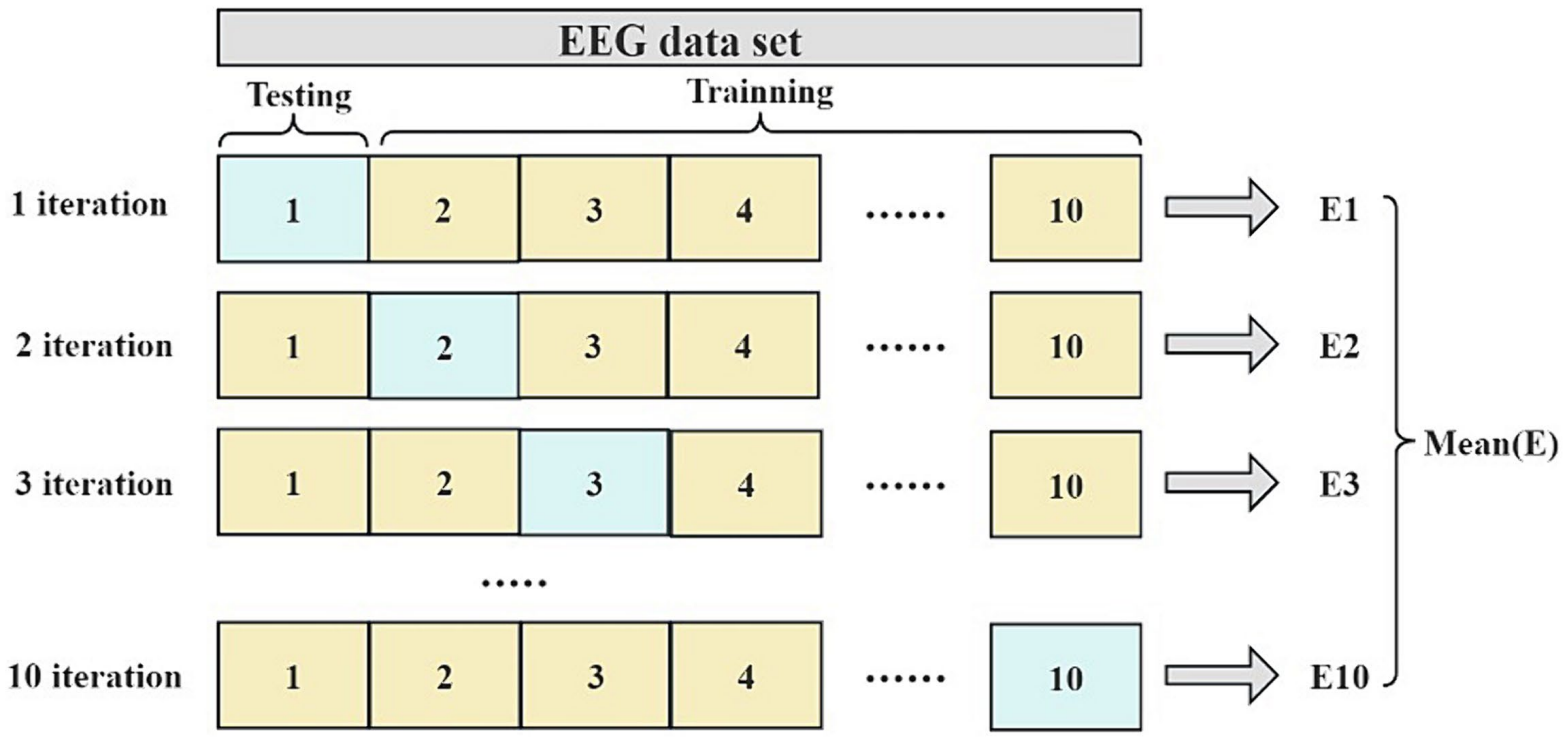
The flowchart of the 10-fold cross-validation.

This paper evaluates the generalization ability of the classification models using a 10-fold cross-validation, a typical statistical method for assessing machine learning models’ generalization capability. This method is particularly useful in situations where limited data is available for model evaluation. In 10-fold cross-validation, the division ratio of training and test sets is consistent, with each fold involving a 90% training data and 10% test data. Upon completing 10 iterations, an array of performance metrics is obtained, and their average is calculated to gauge the overall model performance. Figure 2 illustrates the process of the 10-fold cross-validation.

#### 2.2.3 CDGL classification method

**Figure 3:**
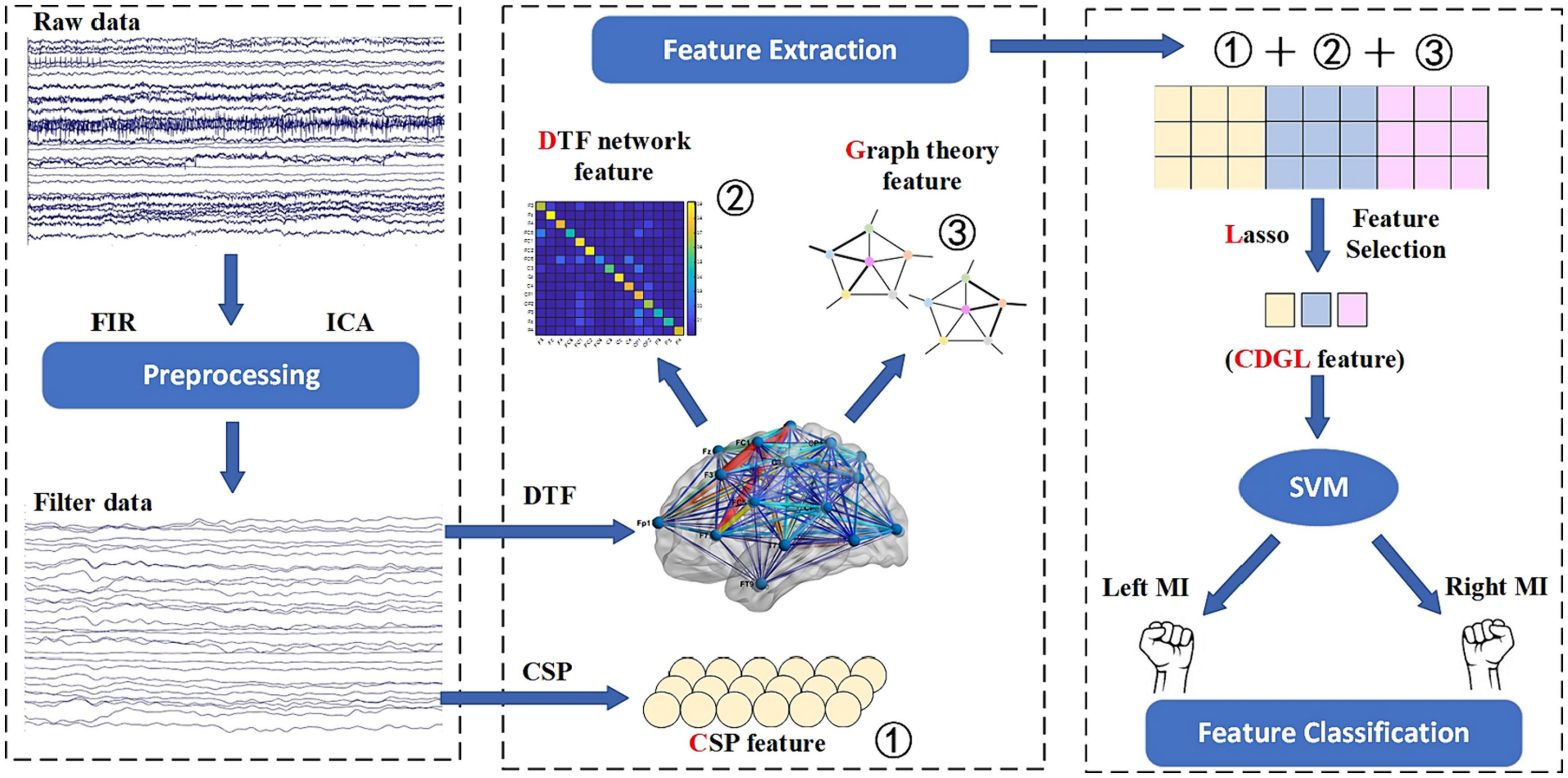
The flowchart of the CDGL method.

A method incorporating CSP, DTF, graph theory feature, and Lasso regularization (CDGL) is proposed in this paper, which innovatively integrates five features with the aim of enhancing classification accuracy in MI-BCI applications. The choices of CSP, DTF, ND, CC, and GE features were made in order to provide a comprehensive representation of brain activity. In this study, these five features were initially combined, and subsequently selected by a Lasso method to eliminate redundant features. The resulting integrated features were then fed into the SVM classifier. Each of these features adds a unique dimension to the analysis, facilitating the exploration of different temporal, spatial, frequency, and connective information of EEG signals. The amalgamation of these diverse features significantly enhances the robustness and accuracy of the SVM classifier. The flowchart of the CDGL method is shown in Figure 3.

To assess the efficacy of the CDGL algorithm, this study conducts a comparative analysis with EEGNet, a widely recognized deep learning algorithm for EEG signal processing. EEGNet is a specialized lightweight convolutional neural network, which is tailored for EEG signal processing. Its architecture encompasses standard convolutional layers, depthwise convolutional layers, and separable convolutional layers, which can integrate spatial and temporal features effectively. This integration renders EEGNet adept at various EEG analysis tasks. This paper adopted a standard architecture of EEGNet, consistent with the framework presented in the reference literature (Lawhern et al., 2018), without any modifications. The training and testing sets were divided into 80 and 20%, respectively. The loss function was chosen to be *Cross Entropy Loss*, and the optimizer selected was *Adam*. The number of epochs was set as 100, and the batch size was set as 16. The ‘kernLength’ was set to 32, and the dropout rate was established at 0.5. The dimension of input for EEGNet was # of trials × # of channels × sampling time, where # of channels was set to 4 or 8.

### 2.3 Evaluating metric

Three evaluation metrics: accuracy, sensitivity, and specificity, are primarily utilized in this paper for testing classification results. These metrics provide a comprehensive framework for assessing model performance.

(1) The calculation of accuracy is shown in Eq. 2.21.


(2.21) $$\mathrm{accuracy}=\frac{TP+TN}{TP+TN+FP+FN}\text{,}$$

where *TP* is the number of samples that are actually positive and have been classified as positive by the classifier. *TN* is the number of samples that are actually negative and have been classified as negative by the classifier. *FP* is the number of samples that are actually negative but have been classified as positive by the classifier. *FN* is the number of samples that are actually positive but have been classified as negative by the classifier. Accuracy is the most intuitive performance metric, representing the overall proportion of correct predictions for both positive and negative classes by the model.

(2) The calculation of sensitivity is shown in Eq. 2.22.


(2.22) $$\mathrm{sensitivity}=\frac{TP}{TP+FN}\text{,}$$

where sensitivity is a measure of a classifier’s ability to correctly identify positive samples, with the advantage of being able to accurately capture positive samples.

(3) The calculation of specificity is shown in Eq. 2.23.


(2.23) $$\mathrm{specificity}=\frac{TN}{TN+FP}\text{,}$$

where is a measure of a classifier’s ability to correctly identify negative samples, enabling accurate exclusion of these instances and reducing false positives.

### 2.4 Statistical analysis methods

In this study, ANOVA and dependent sample *t*-test are used as the statistical analysis methods. To avoid errors associated with repeated measurements, the Bonferroni correction method is also employed here. Specifically, if five comparisons were made, the significance level was adjusted from the nominal α = 0.05 to α = 0.01 (0.05/5), thus maintaining a very stringent criteria for statistical significance. In ANOVA, this study employed both one-way ANOVA and two-way ANOVA. The two-way ANOVA was used to compare the effects of two factors on the experimental outcomes, as well as to determine whether there is an interaction effect between these factors. The ANOVA can analyze the significant changes of means across multiple groups effectively, whereas the dependent sample *t*-test is more suitable for comparing means within the same group under varying conditions, offering sensitivity for detecting changes within the group. Actually, this paper performed the within-subjects test. To demonstrate the authenticity of the study, this research utilizes a paired *t-*test and Bonferroni correction method on a public dataset to discern the notable differences between CDGL and EEGNet.

To be aligned with the research objectives, this study organized the statistical analysis methods. For objective 1, one-way ANOVA, two-way ANOVA, and paired *t*-test were employed to analyze and compare the capabilities of different features in classifying MI-EEG data. For objective 2, one-way ANOVA was used to assess the effect of feature combinations and to investigate the impact of the number of channels and frequency bands. Additionally, paired *t*-tests and Bonferroni correction methods were utilized to validate the differences between CDGL and EEGNet on public datasets.

### 2.5 Data acquisition and preprocessing

#### 2.5.1 Data acquisition instructions

**Figure 4:**
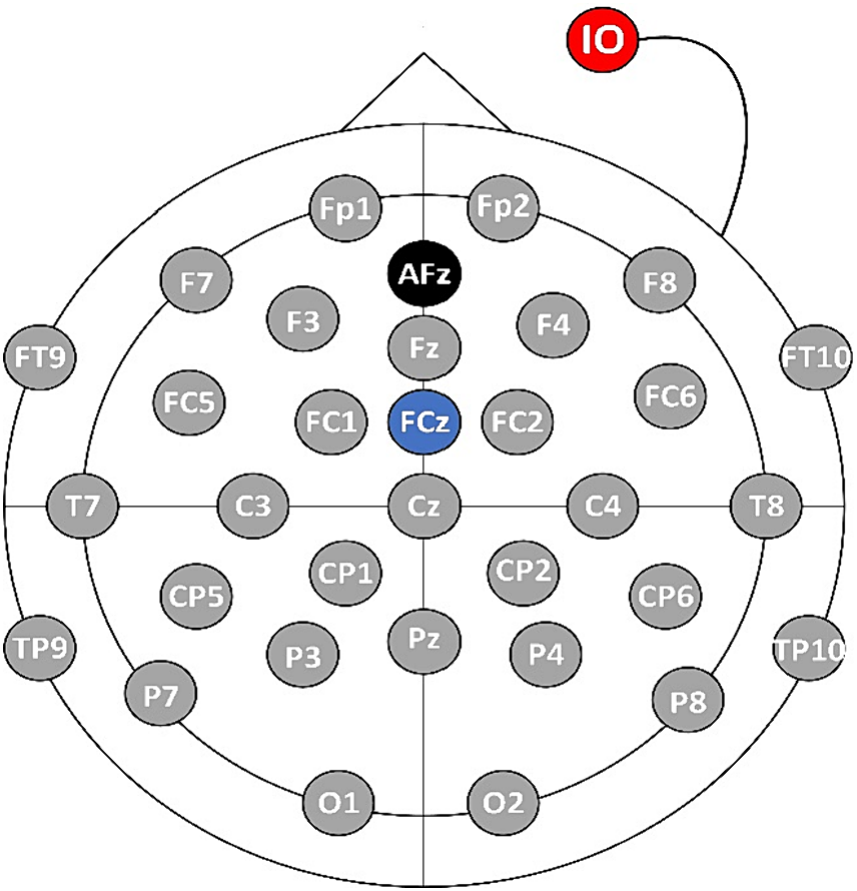
The distribution diagram of electrode positions.

In this paper, an EEG signal acquisition experiment was conducted on a MI tasks involving 26 subjects aged between 23 and 27. The MI task is described as the mental simulation of hand grasping action being performed by an individual without the actual execution of the action. In the experiment, a 32-lead EEG equipment from Brain Products (BP) Inc. was used to collect EEG data from the MI BCI, and the sampling frequency was set at 500 Hz. Prior to the experiment, various steps were taken to ensure the suitability of the subjects. Firstly, all subjects underwent vision correction to ensure normal visual acuity. Additionally, a thorough examination was conducted to verify their mental health and overall well-being. The subjects were informed about the purpose and significance of the experiment beforehand. Following this, the subjects wore an EEG cap and were seated in front of a computer as instructed by the experimenter. The experimenter applied the conductive paste to the EEG cap, reducing the resistance to less than 5 kΩ. Throughout the experimental period, the subjects were instructed to maintain a stable mental state and avoid intense emotional fluctuations, ensuring data integrity. The electrode positions were set based on the international 10–20 lead standard, as shown in Figure 4. The AFz electrode (marked in black in Figure 4) serves as the ground electrode, and the FCz electrode (marked in blue in Figure 4) is used as the reference electrode, and the IO electrode (marked in red Figure 4) is used as the Electrooculography electrode.

**Figure 5:**
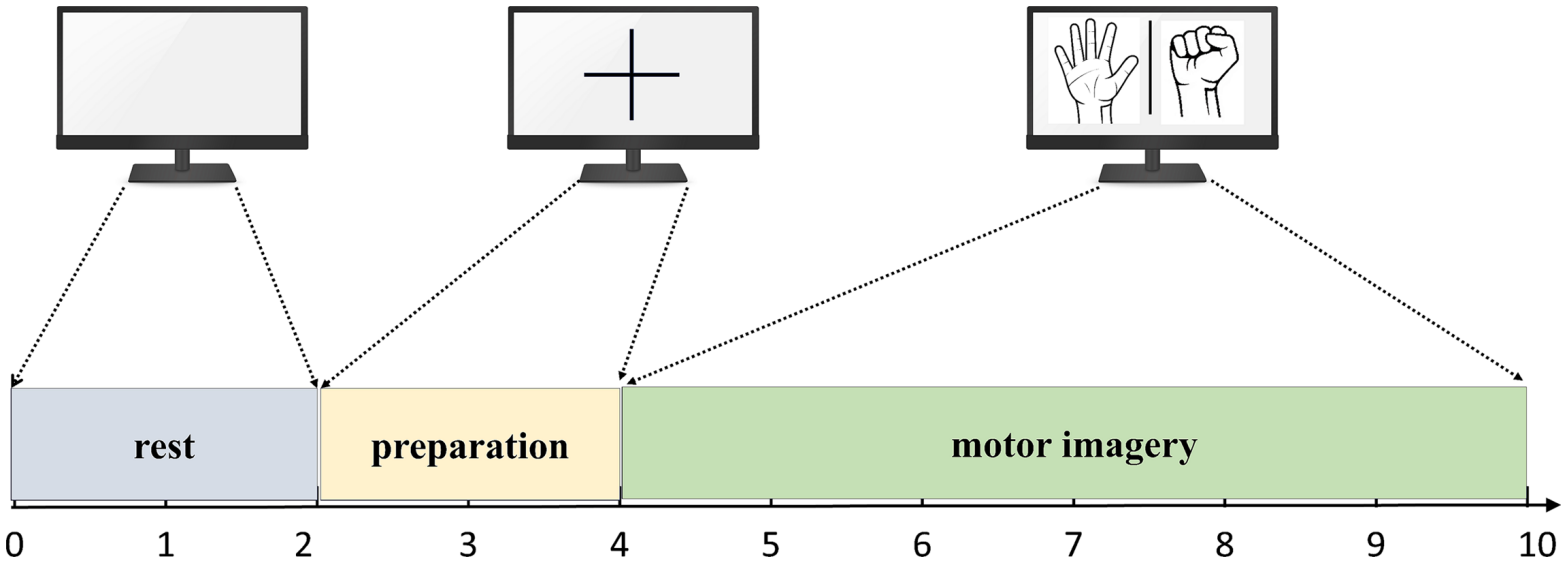
The flow of the MI experiment.

During the acquisition process, the subjects performed corresponding tasks based on the interface displayed on the computer screen. Each experiment had a duration of 10 s, consisting of different stages. Firstly, there was a 2-s period where the screen would display a blank interface, and subjects were expected to be in a relaxed state. Following this, a 2-s period followed where a cross interface appeared, indicating the preparation state for the MI tasks. Lastly, the screen displayed either a left hand fist or a right hand fist for 6 s, during which subjects were required to carry out the MI tasks corresponding to the displayed hand. The flow of the experiment is summarized in Figure 5.

Each subject performed two sets of experiments, one set of experiments performed a left hand MI tasks 40 times, and one set of experiments performed a right hand MI tasks for a total of 80 experiments, obtaining EEG data in the shape of 32*3000*80 (32 represents the number of EEG channels, 3,000 represents the data length sampled over 6 s at a sampling frequency of 500 Hz, and 80 represents the number of trials).

#### 2.5.2 Data preprocessing

EEG signal preprocessing is a crucial process that involves applying a series of steps to raw EEG data. The goal is to purify the signal, eliminate noise, and prepare the data for subsequent analysis. Pure EEG signals are of crucial importance for accurate analysis. In the pre-processing phase, an 8 Hz to 40 Hz bandpass filter was applied to extract the study’s key frequency components and eliminate potential low-frequency artifacts and high-frequency noises. With a Finite Impulse Response (FIR) design, the filter was configurated for achieving its linear phase response, effectively preventing a phase distortion. The filter’s order was algorithmically determined, based on a predefined multiple of the sampling rate and the lower cutoff frequency. Post-filtering, the Independent Component Analysis (ICA) algorithm from EEGLAB (Delorme and Makeig, 2004) was employed to eliminate artifacts, including eye movements and muscle activity, resulting in purified EEG signals. To ensure the validity of MI, a time window of 3 s was selected for analysis.

#### 2.5.3 Public datasets

To validate the effectiveness of the proposed algorithm, this study employs the datasets from BCI Competition IV 2a (Tangermann et al., 2012) and PhysioNet’s BCI2000 (Schalk et al., 2004). The BCI IV 2a dataset records EEG data through 22 scalp electrodes at a 250 Hz sampling frequency. In the experiment, each subject performed 6 experimental runs, totaling 48 trials (12 each for left-hand, right-hand, both feet, and tongue MI). The average duration of each trial was approximately 8 s, with an actual MI period of 3 s. On the other hand, the BCI2000 dataset employs 64 scalp electrodes and captures data at a 160 Hz sampling frequency, featuring eight tasks that include MI of the left hand, right hand, both hands, both feet, and actual movement tasks. Each subject performed 14 experimental runs, totaling 84 trials. Each trial lasted for 4 s. The MI task studied in this article is a binary classification task with a left-hand MI class and a right-hand MI class. To ensure consistency with our experimental tasks, the same electrodes and MI tasks (left and right hand) were also selected from these public datasets.

## 3 Results

### 3.1 Data collected by IMUT

#### 3.1.1 The effect of CSP, DTF, graph theory features on MI task classification performance

To investigate the effect of network features on the classification performance of MI tasks, a DTF brain network model was constructed using various channel configurations, including 4-channel (FC1, FC2, C3, and C4), 8-channel (Fz, FC1, FC2, C3, Cz, C4, CP1, and CP2), 12-channel (Fz, FC1, FC2, C3, Cz, C4, CP1, CP2, F3, F4, P3, and P4), 15-channel (Fz, FC1, FC2, C3, Cz, C4, CP1, CP2, F3, F4, P3, P4, FC5, and FC6), and 32-channel configurations. The actual values of DTF matrices are used here to construct the feature set.

The DTF coefficient connection matrix was selected as the feature set for the classification task, and a SVM was employed as the classifier. To ensure the stability of the classification results, a 10-fold cross-validation method was utilized. The study involved 26 participants who performed MI classification task in both the Alpha and Beta frequency bands. The average classification performance of the DTF + SVM method is presented in Table 1.

**Table 1:** The average classification performance of the DTF + SVM method using different channel configurations for 26 subjects.

	Accuracy				Sensitivity				Specificity			
	Alpha		Beta		Alpha		Beta		Alpha		Beta	
	Mean	*SD*	Mean	*SD*	Mean	*SD*	Mean	*SD*	Mean	*SD*	Mean	*SD*
Channels = 4	72.03	7.9	75.70	8.9	74.33	9.6	76.15	9.1	69.72	9.9	76.44	10.8
Channels = 8	76.03	9.1	81.14	8.7	77.12	9.2	81.48	9.2	75.69	9.8	79.81	10.2
Channels = 12	79.65	8.3	84.30	7.8	80.71	8.6	84.39	7.7	78.01	10.9	83.91	9.7
Channels = 15	82.43	7.8	85.54	9.3	83.30	8.1	86.73	7.5	80.33	11.2	85.45	9.5
Channels = 32	89.17	6.7	91.74	5.9	90.30	7.6	92.32	6.5	87.12	8.8	90.51	8.3

Table 1 demonstrates that DTF network features possess the capability to distinguish between left and right hand MI tasks, enabling accurate recognition of these tasks. It was observed that as the number of channels increased, the classification accuracy also improved. Notably, when utilizing 32 channels, the classification system not only reached a high level of accuracy at 91.74%, but also demonstrated a sensitivity of 92.32% and a specificity of 90.51%. Furthermore, analysis of the Alpha and Beta bands revealed that the DTF coefficient matrix yielded slightly higher classification accuracy for the Beta band compared to the Alpha band. In this study, a Two-Factor Analysis of Variance (ANOVA) was performed to provide a detailed analysis of the results. This analysis is crucial for evaluating the effects of various channel combinations (X1) and frequency band (X2) analyses on the essential metrics of the research.

Table 2 displays the results of ANOVA analyses, which provide a comparative overview of accuracy, specificity, and sensitivity under different conditions. The results indicated significant effects of channel combinations on the three metrics: accuracy [*F*(4, 250) = 33.16, *p* < 0.0001], specificity [*F*(4, 250) = 28.35, *p* < 0.0001], and sensitivity [*F*(4, 250) = 19.89, *p* < 0.0001], each showing considerable differences. Similarly, frequency band types significantly influenced these metrics, as shown by accuracy [*F*(1, 250) = 14.73, *p* = 0.000157], specificity [*F*(1, 250) = 17.47, *p* < 0.0001], and sensitivity [*F*(1, 250) = 16.05, *p* < 0.0001]. However, no significant interaction effect was observed between channel combination and frequency band [Accuracy: *F*(4, 250) = 0.17, *p* = 0.9546; Specificity: *F*(4, 250) = 0.27, *p* = 0.8941; Sensitivity: *F*(4, 250) = 0.28, *p* = 0.8881], suggesting that the interaction of channel combination and frequency band type does not significantly alter these outcomes. BrainNet Viewer (Xia et al., 2013) software was employed to visualize the connectivity matrix. It is worth noting that IO, TP9, and TP10 electrodes were excluded from the visualization due to channel position considerations.

**Table 2:** The comparison of accuracy, specificity, and sensitivity for DTF+SVM using a two-way ANOVA analysis.

	Accuracy		Sensitivity		Specificity	
	*F*	*P*	*F*	*P*	*F*	*P*
X1	33.16	3.46e-22	28.35	1.51e-19	19.89	3.07e-14
X2	17.47	4.03e-5	11.57	7.79e-4	16.05	8.12e-5
X1*X2	0.1678	0.9546	0.2714	0.8941	0.2842	0.8881

**Figure 6:**
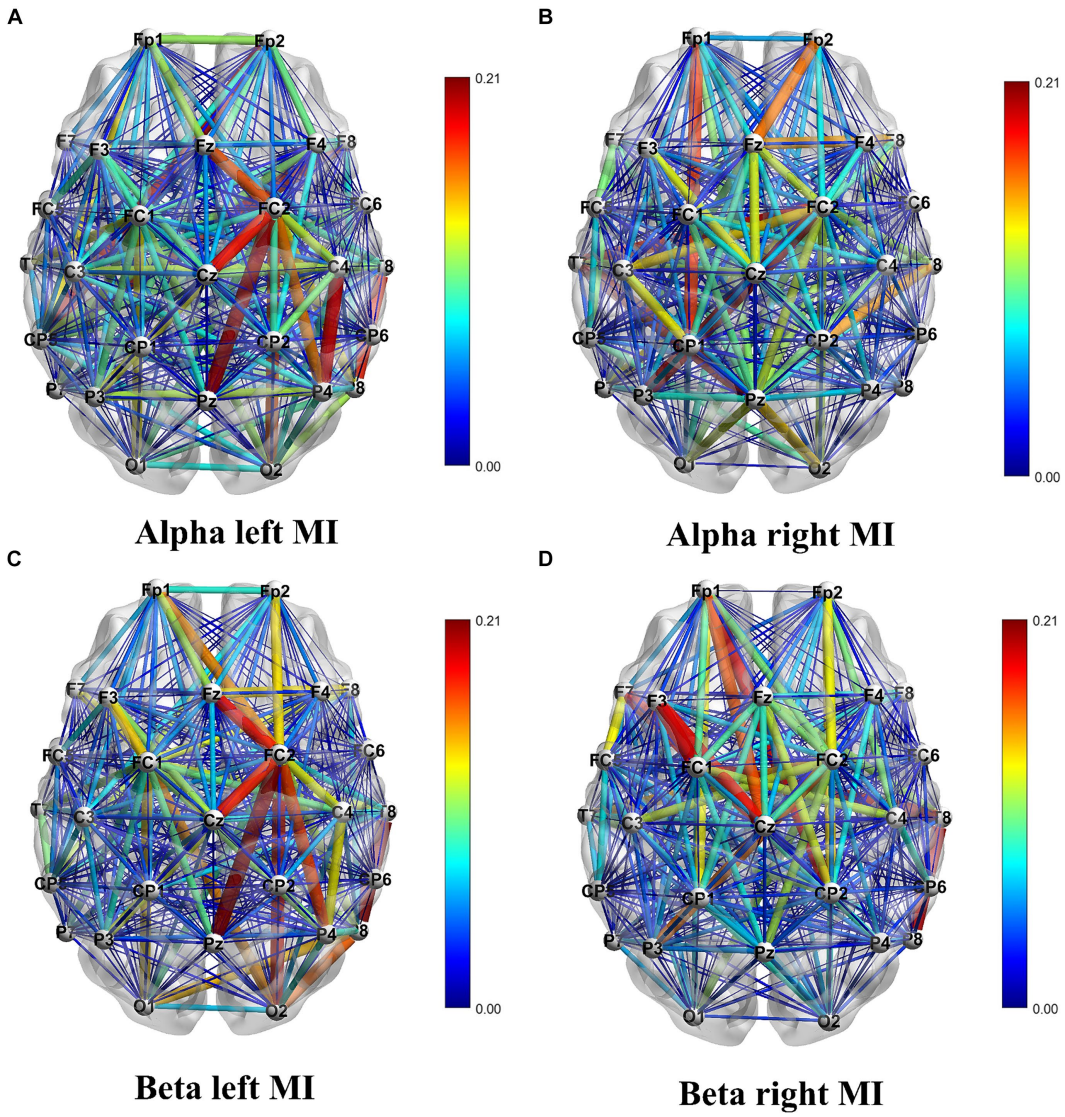
Visualization of the DTF connectivity matrix. Panel **(A)** shows the DTF connectivity matrix for left hand MI in the Alpha frequency band. Panel **(B)** shows the DTF connectivity matrix for right hand MI in the Alpha frequency band. Panel **(C)** shows the DTF connectivity matrix for left hand MI in the Beta frequency band. Panel **(D)** shows the DTF connectivity matrix for right hand MI in the Beta frequency band.

The analysis of Figure 6 reveals a distinction in the direction of EEG signal transmission during MI tasks involving different hands. The color gradient from blue to red signifies weaker to stronger connections, respectively. This difference is a reliable foundation for accurately classifying left and right hands movements. When performing MI tasks with the left and right hands, the information connectivity patterns within the brain regions exhibit significant variations. To be more specific, there is an increase in connectivity strength within the right hemisphere of the brain when engaging in MI of the left hand, and conversely, an increase within the left hemisphere when MI of the right hand.

The DTF graph-theoretic features serve to depict the information transfer relationships between different brain regions by transforming the DTF coefficient connectivity matrix into graph structures and extracting relevant features. The topology of the brain network is represented by feature vectors derived from three graph theory features: node degree (ND), clustering coefficient (CC), and global efficiency (GE). This paper directly utilizes the DTF coefficient matrix as a directed weighted graph for calculating the graph-theoretic features. Notably, ND assumes a pivotal role in graph theory analysis as it quantifies the significance or activity level of individual nodes within the network.

**Figure 7:**
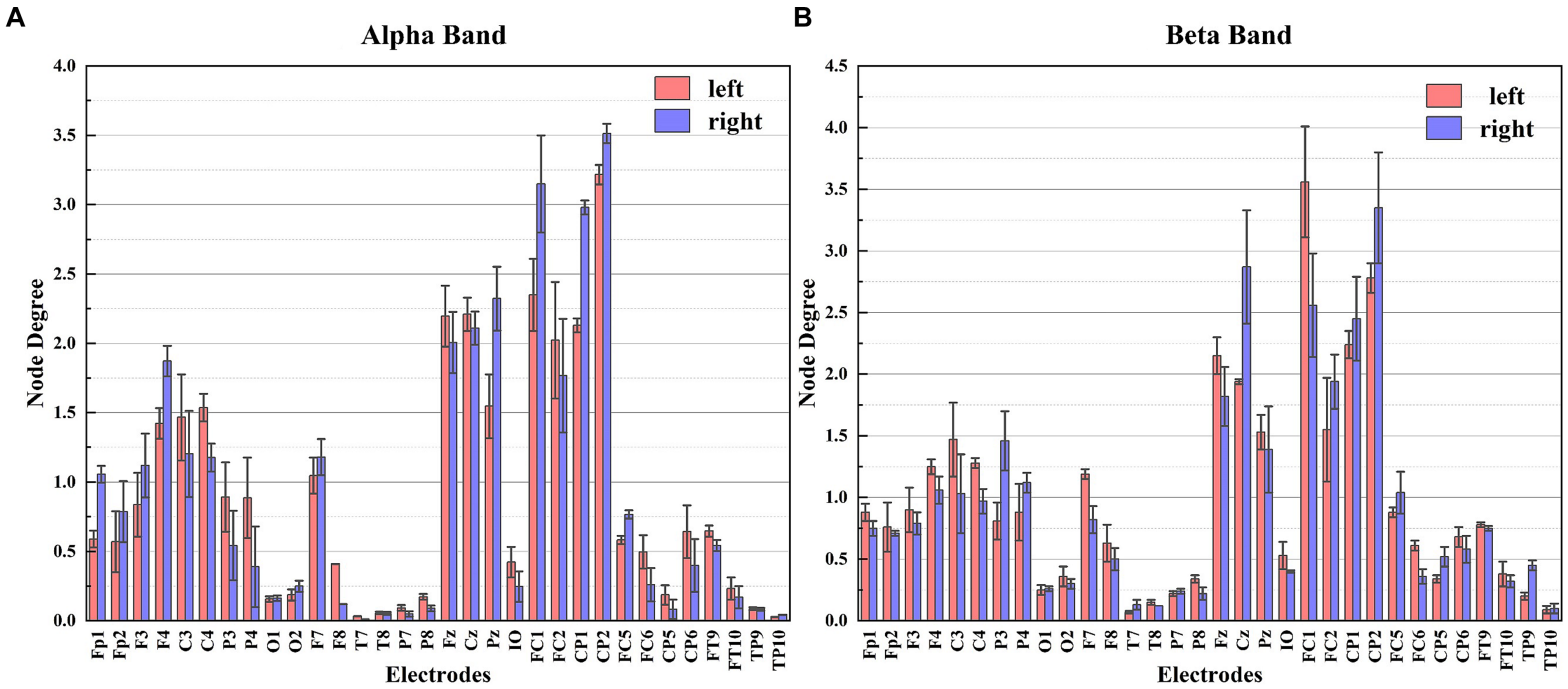
The node degree of each electrode for different frequency bands. Panel **(A)** shows the node degree of each electrode in the Alpha frequency band. Panel **(B)** shows the node degree of each electrode in the Beta frequency band.

Based on the ND in Figure 7, notable distinctions in ND are observed among the electrodes during the two different MI tasks, particularly for the C3, C4, Cz, P3, P4, FC1, and FC2 electrodes. These discrepancies highlight the regions of the brain where these electrodes are positioned, which exhibit significant information flow and strong connectivity with other electrodes during MI tasks. Taking this characteristic as a feature in the classification of MI tasks proves effective in accurately distinguishing between left and right hand MI tasks.

**Figure 8:**
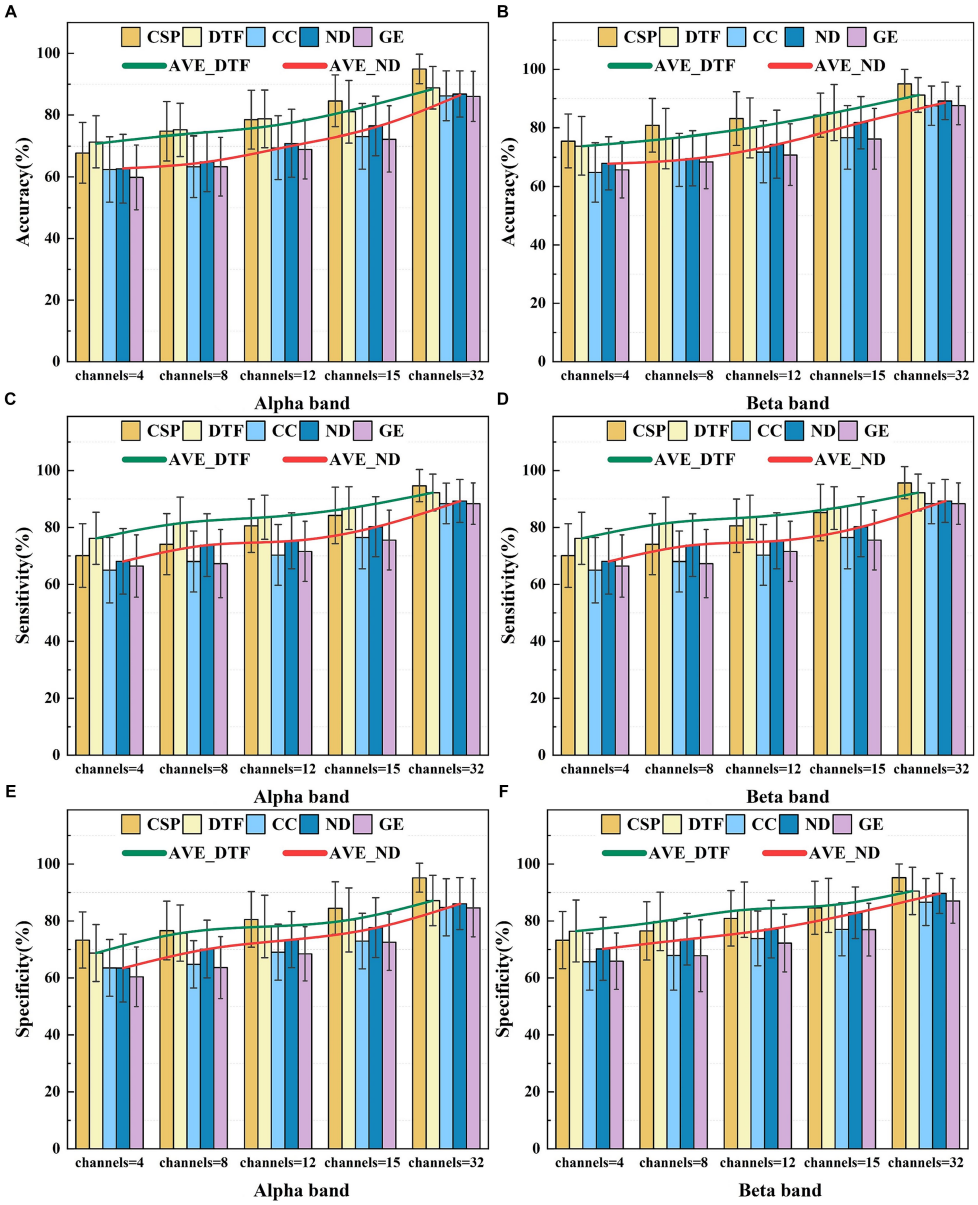
The average classification performance of five feature selections for different frequency bands. Panel **(A)** shows the average classification accuracy in the Alpha frequency band. Panel **(B)** shows the average classification accuracy in the Beta frequency band. Panel **(C)** shows the average classification sensitivity in the Alpha frequency band. Panel **(D)** shows the average classification sensitivity in the Beta frequency band. Panel **(E)** shows the average classification specificity in the Alpha frequency band. Panel **(F)** shows the average classification specificity in the Beta frequency band.

Figure 8 illustrates the average performance achieved using an SVM classifier for five distinct features of EEG signals extracted from 26 subjects. These features include CSP features, DTF network features, ND features, CC features, and GE features.

Analysis of Figure 8 reveals how the number of electrodes influences complexity of the brain functional network and the discriminative ability of feature selections. As the number of electrodes increases, the classification accuracy using the graph theory features for the MI-BCI tasks demonstrates an increasing trend [confirmed by a one-way ANOVA under Alpha for ND feature, *F*(4, 250) = 2.95, *p* < 0.001]. In the Alpha band, utilizing 32 channels, ND features exhibited average classification accuracy, sensitivity, and specificity of 86.88, 87.54, and 86.06%. CC features showed values of 85.22, 87.61, and 84.75%, while GE features had 86.04, 87.17, and 84.62%. In the Beta band, ND features demonstrated corresponding values of 87.51, 89.33, and 89.64%; CC features presented 89.15, 88.38, and 86.59%; and GE features had 87.57, 88.35, and 87.08%, respectively. ND’s performance between Alpha and Beta frequency bands revealed significant differences by one-way ANOVA in the results of *F*(1, 50) = 4.19, *p* = 0.04 for accuracy, *F*(1, 50) = 4.08, *p* = 0.04 for sensitivity, and *F*(1, 50) = 4.67, *p* = 0.03 for specificity. CC’s performance: *F*(1, 50) = 4.61, *p* = 0.03; *F*(1, 50) = 4.94, *p* = 0.03; *F*(1, 50) = 4.6, *p* = 0.03 for the three metrics, respectively. GE’s performance: *F*(1, 50) = 4.16, *p* = 0.04; *F*(1, 50) = 4.19, *p* = 0.04; *F*(1, 50) = 4.23, *p* = 0.04 for the three metrics, respectively. However, due to feature redundancy, the effectiveness of these graph theory features on classification tasks remains slightly lower than the performance of the traditional CSP algorithm, which achieves an accuracy of 94.91% [In Beta: ANOVA in the results of *F*(1, 50) = 12, *p* < 0.001 for ND feature, *F*(1, 50) = 19.82, *p* < 0.001 for CC feature, and *F*(1, 50) = 19.22, *p* < 0.001 for GE feature].

#### 3.1.2 The effect of feature fusion on MI task classification performance (including the new method proposed)

**Figure 9:**
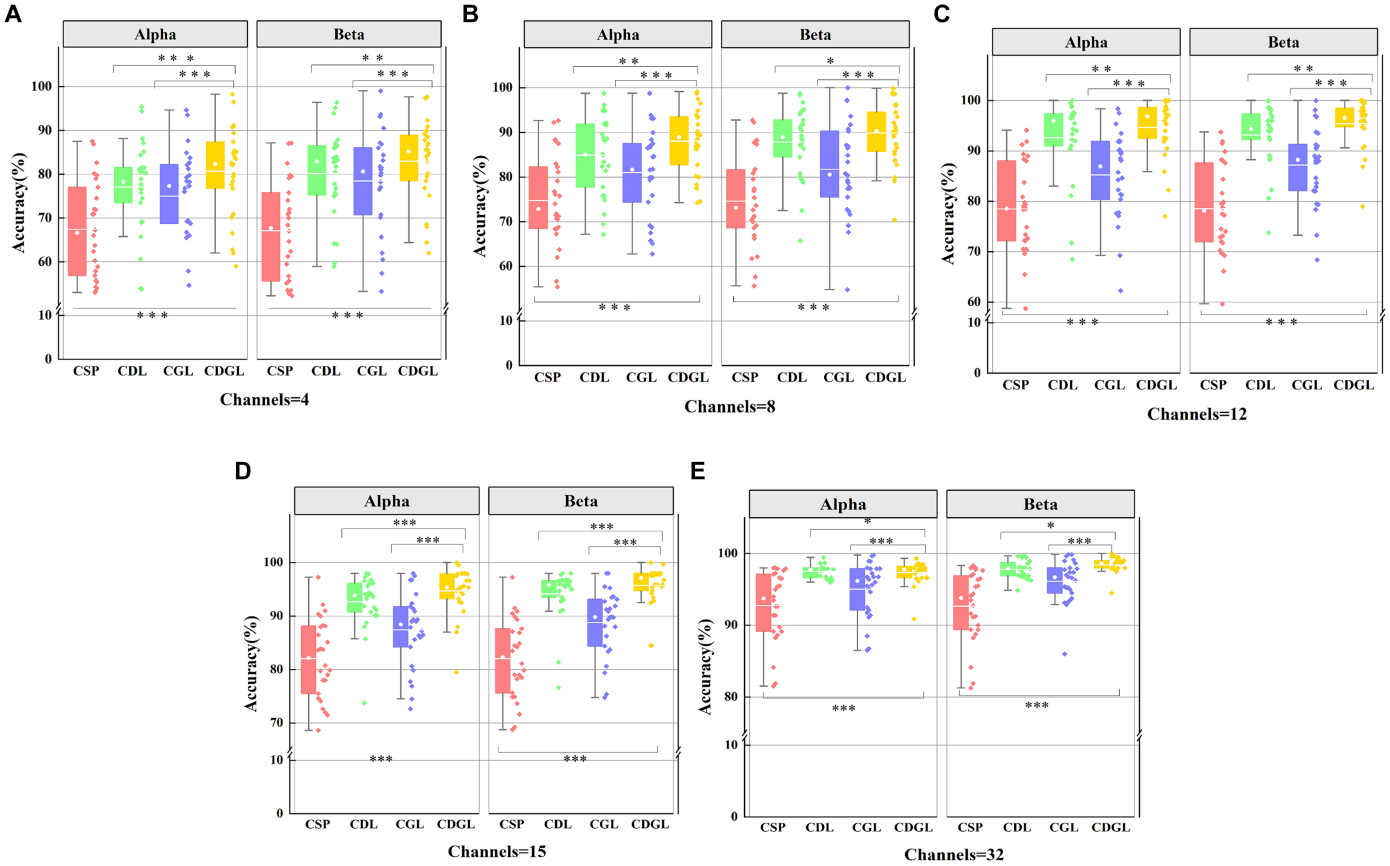
The classification accuracies of four methods for two frequency bands with five different channel combinations. Panel **(A)** shows the classification accuracy for two frequency band and 4 channels. Panel **(B)** shows the classification accuracy for two frequency band and 8 channels. Panel **(C)** shows the classification sensitivity for two frequency band and 12 channels. Panel **(D)** shows the classification sensitivity for two frequency band and 15 channels. Panel **(E)** shows the classification specificity for two frequency band and 32 channels.

The CSP algorithm is effective in extracting spatial features from EEG signals. However, since the brain exhibits time-varying characteristics during MI tasks, a single spatial feature cannot fully capture all the information relating to the left and the right hand MI. To address this limitation, the DTF network features and graph theory features are integrated into the CSP algorithm to explore the impact of the DTF brain functional network on the classification effectiveness of MI tasks. As the fused features possess high dimensionality, they are susceptible to feature redundancy, which can lead to a decrease in the classification accuracy. To avoid this issue, the Lasso method is employed to screen the fused features, selecting the optimal ones for classification. Figure 9 presents box-and-line plots for four MI EEG decoding algorithms under two frequency bands: the CSP algorithm, the CSP added DTF with Lasso regularization (CDL), the CSP added graph theory with Lasso regularization (CGL), and CSP added DTF and graph theory with Lasso regularization (CDGL). Due to the relatively balance between different classes for the datasets, only one metric “accuracy” is chosen to evaluate the algorithm performance.

Figure 9 reveals that the accuracy of CSP algorithms can be enhanced by integrating DTF network features and graph theory features into traditional CSP algorithms. As the number of channels increases, all four algorithms’ average classification accuracy improves. Notably, both the CDL, CGL, and CDGL algorithms outperform the traditional CSP algorithms. A One-way ANOVA was used here to test for significance, yielding a result of *F*(3, 100) = 15.12, *p* < 0.001. Following this result, a *post hoc* analysis was further conducted using the “*multcompare*” function in MATLAB, with a “*CType*” parameter set to “*tukey–kramer*,” which means the Tukey HSD method was used. The analysis indicated that there are significant differences between the CSP algorithm and the other three algorithms, with all *p*-values being less than 0.005. When the number of channels reaches 32, the CDGL algorithm achieves higher accuracy in the Beta frequency band than in the Alpha frequency band [confirmed by a one-way ANOVA, *F*(1, 50) = 4.55, *p* = 0.03]. This observation suggests that the Beta band exhibits more intricate and diverse signal features, which may be attributed to highlight brain activity and enhanced information processing capacity during cognitive tasks. Here, in Figure 9, one single asterisk (*) indicates a significance level of 0.05, double asterisks (**) indicates 0.01 level, and triple asterisks (***) indicates 0.001 level.

### 3.2 Data from BCI competition and PhysioNet BCI2000

#### 3.2.1 The effect of CSP, DTF, graph theory features on MI task classification performance

To validate the aim drawn in this paper, the algorithms discussed in this article were also tested using the BCI Competition IV 2a dataset and PhysioNet’s BCI2000 dataset. To maintain data consistency, the validation was conducted using the same electrodes, specifically the 4-channel and 8-channel configurations. The broken lines depict the accuracy, sensitivity, and specificity metrics of the six classification methods according to different channels and different frequency band. Panels (a), (b), and (c) present the results obtained from the BCI IV 2a dataset, whereas panels (d), (e), and (f) show the results from PhysioNet’s BCI2000 dataset.

**Figure 10:**
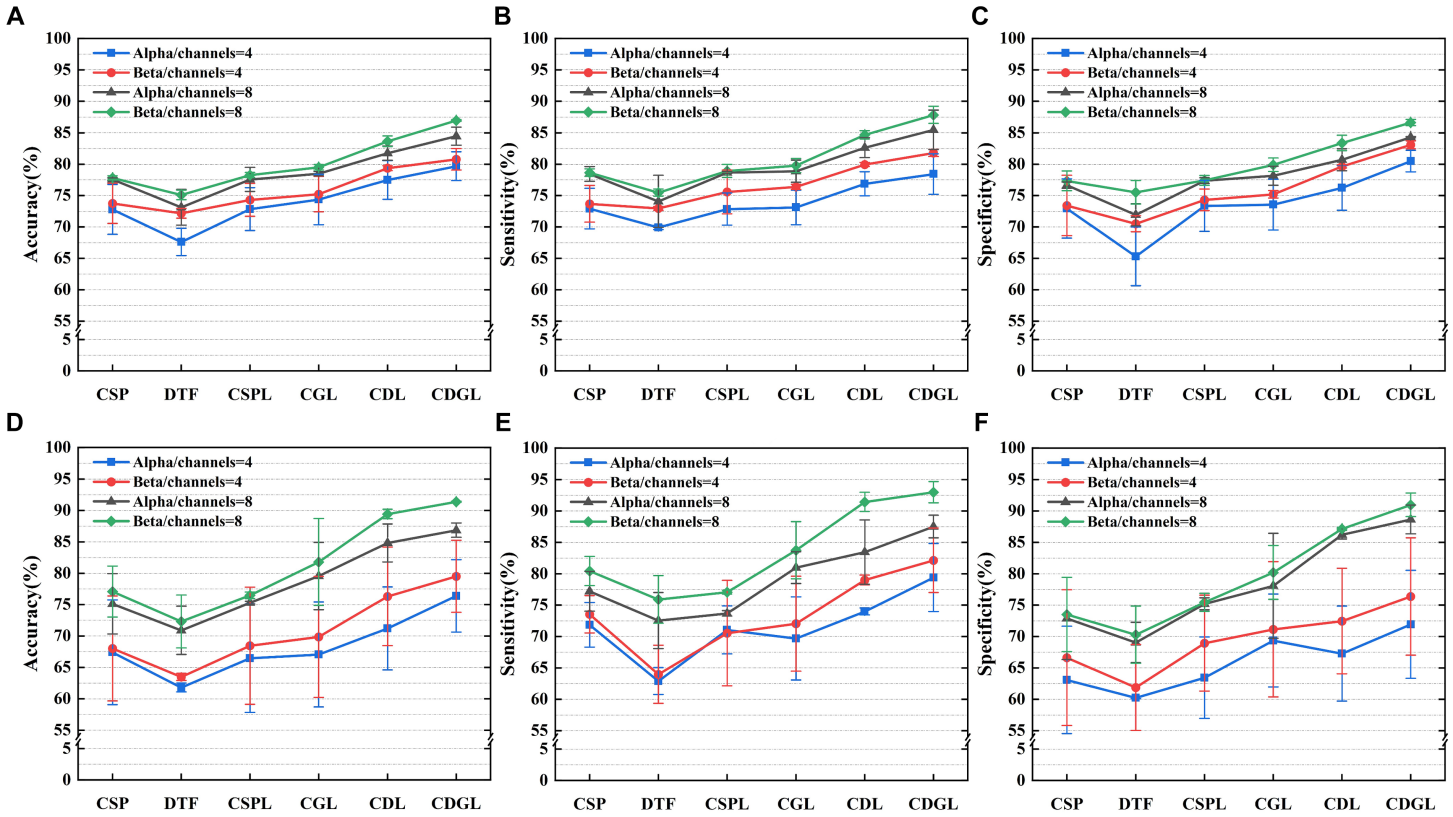
Three metrics of the six algorithms for the MI-BCI task classifications for two frequency bands and the two different datasets. Panels **(A–C)** display the classification accuracy, sensitivity, and specificity, respectively, for the BCI competition dataset. Panels **(D–F)** display the classification accuracy, sensitivity, and specificity, respectively, for the PhysioNet BCI2000 dataset.

It can be easily seen from Figure 10 that the algorithms involved in 4-channel are less correctly classified than 8-channel, both in the Alpha band and in the Beta band. The accuracy, sensitivity, and specificity of CDGL (CSP+DTF+Graph theory feature+Lasso) are significantly higher than that of CSP, DTF, CSPL (CSP+Lasso), CDL (CSP+DTF+Lasso), and CGL (CSP+Graph theory feature+Lasso). As the analysis focuses on discerning significant differences among various algorithms applied to the same datasets, a paired sample *t*-test is employed to ascertain the statistical disparities between the CDGL algorithm and others. Due to requiring multiple comparisons, therefore, the Bonferroni correction was employed, leading to the adjustment of the *p*-value from 0.05 to 0.01 (0.05/5). The findings reveal that the *p*-values of the three assessment model indicators are consistently below the significance threshold of 0.01, whether within the BCI IV 2a dataset or the PhysioNet’s BCI2000 dataset.

#### 3.2.2 The effect of feature fusion on MI task classification performance (including the new method proposed) and comparison with EEGNet method

In the validation using public datasets, this paper also explored the impact of the feature fusion algorithm CDGL on classification performance. As shown in Figure 10, the CDGL algorithm’s performance was rigorously evaluated under varying conditions of channel and frequency band configurations using the three specific evaluation metrics. Comparisons were conducted in two primary scenarios: First, the algorithm’s performance were compared between alpha band and beta band while maintaining constant channel settings. This involved an assessment of the performance in alpha and beta bands separately configured at channels = 4 or channels = 8. Secondly, the study focused on comparing the algorithm’s performance metrics across different channel configurations, channels = 4 and channels = 8, within unchanged frequency band. The results of each comparison show significant differences (all *p*-value <0.0125). This comparative analysis was aimed at exploring the impact of channel and frequency band variations on the effectiveness of the CDGL algorithm.

**Figure 11:**
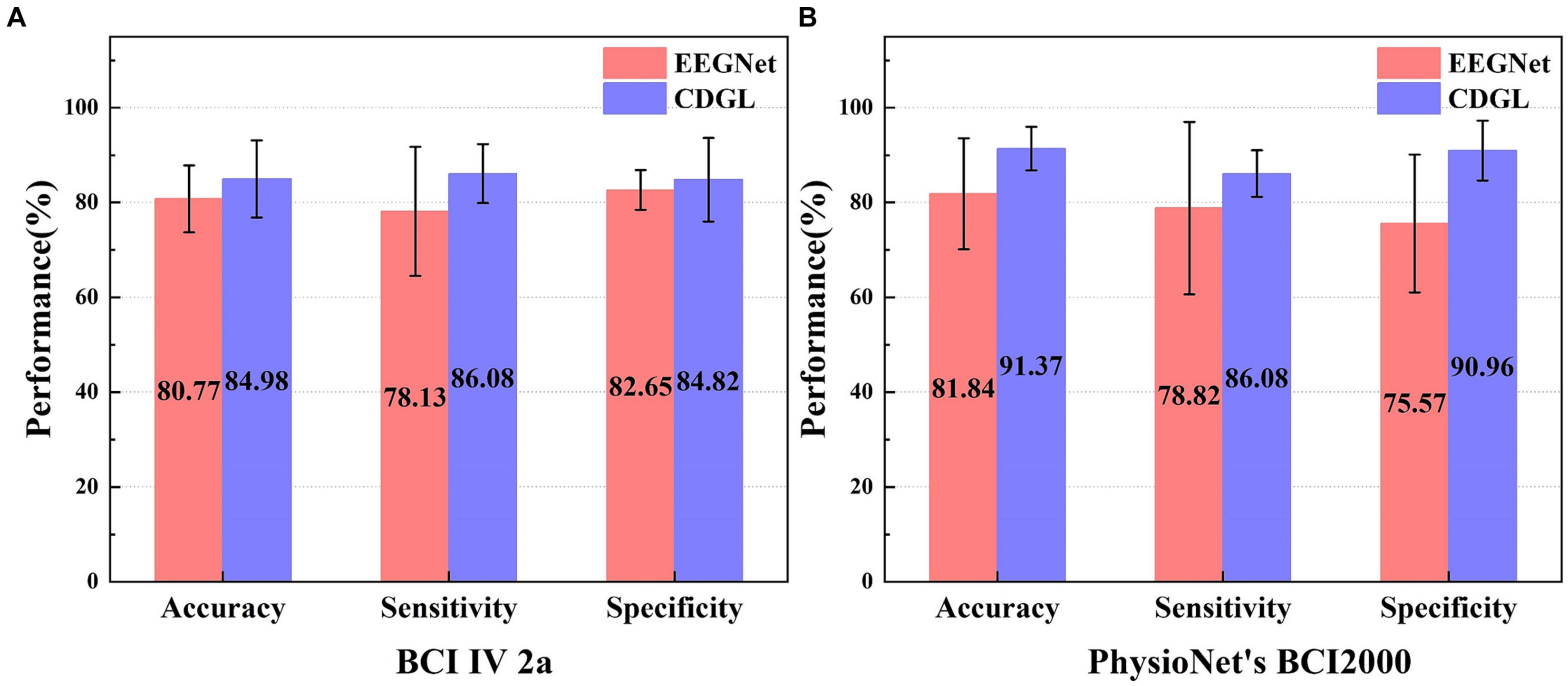
The performance comparison of two algorithms (EEGNet and CDGL) on two different datasets (BCI IV 2a and PhysioNet’s BCI2000). Panel **(A)** shows the classification performance of two algorithms for the BCI IV 2A dataset. Panel **(B)** shows the classification performance of two algorithms for the PhysioNet BCI2000 dataset.

In addition, this paper also made a comparison with one of gold-standard methods (EEGNet) commonly used for MI-BCI classification tasks. The comparison results of the three performance indicators of the two algorithms are shown in Figure 11.

The parameter selection for CDGL is as channel numbers = 8, Beta band, the model’s order = 8. A comparative analysis of two models—EEGNet and CDGL—was conducted using the standardized BCI IV 2a and PhysioNet’s BCI2000 dataset. It was indicated that for the three key metrics (accuracy, sensitivity, and specificity), CDGL performs better than EEGNet, affirming the superior capability and dependability of CDGL for the classification of MI-BCI tasks. On the BCI IV 2a dataset, the CDGL achieved 84.98% accuracy, 86.08% sensitivity, and 84.82% specificity respectively, surpassing the values of 80.77, 78.13, and 82.65% reported by EEGNet (Paired *t*-test, *p* = 0.008 for accuracy, *p* = 0.01 for sensitivity and *p* = 0.012 for specificity). Similarly, for the PhysioNet’s BCI2000 dataset, CDGL attained an accuracy of 91.37%, a sensitivity rate of 86.08%, and a specificity rate of 90.96%, in contrast to the 81.84, 78.82, and 75.57% obtained by EEGNet (Paired *t*-test, *p* = 0.012 for accuracy, *p* = 0.014 for sensitivity and *p* = 0.003 for specificity).

## 4 Discussion

This article presents an exploration of a brain functional network construction method based on the DTF. We demonstrate its discriminative ability in left and right hand MI tasks by extracting DTF network features and graph theory features. Specifically, for the left-hand MI tasks, there is a noticeable enhancement in the strength of neural connections within the left hemisphere, indicating an increased neural activity and a significant flow of information (as demonstrated in Figure 6). This increment in activity not only results in reduced energy within the left hemisphere but is also correlated with a decline in power within specific frequency bands (namely, the alpha and beta bands), consistent with the Event-Related Desynchronization (ERD) phenomenon observed in the contralateral sensorimotor cortex. Similarly, this phenomenon occurs within the right hemisphere during right hand MI tasks.

The findings of this study offer a novel approach to enhancing the performance of traditional CSP algorithms. We showed the improved classification results by integrating DTF network features and graph theory features into CSP. These findings are consistent with previous findings (Ghosh et al., 2015; Filho et al., 2018; Li and Zhang, 2022) that indicators of functional brain connectivity have the potential for categorization in the domain of MI tasks. Compared to the methods used in previous research, we innovatively integrate DTF network features with graph theory features, obtaining significantly improved experimental results. Our study further demonstrates the effectiveness of the DTF-based brain functional network construction method for MI tasks. Graph feature application effectively captures the spatial correlations and network structures in EEG data, which are the aspects often overlooked in traditional time-frequency feature analysis. Insight into this spatial correlation is crucial for deeply understanding the brain’s activity patterns while MI tasks. Our use of graph features aims not merely to achieve superior performance on datasets but is based on a profound understanding of brain network analysis and signal processing. Our research delves deeply into the brain activity analysis during the MI process, seeking a comprehensive understanding of these complex activities rather than a mere accumulation of features. It is worth noting that our study was limited to the dichotomous categorization problem for left and right hand MI tasks. Future studies could extend it to more complex multi-categorization MI tasks. This will further validate our proposed approach for various tasks and contexts. In addition, we plan to explore the impact of multiple network feature fusion techniques on motion imagery decoding algorithms. By combining different types of features, such as time-domain features, frequency-domain features, and spatial features, we can expect to further improve the decoding accuracy and robustness of the MI tasks.

The algorithm’s information transmission rate in offline experiments, which utilized 3 s of data, was not enough for a real-time application, for examples brain-controlled wheel chair and robots. The sliding time window sampling might be a reasonable choice to enhance this rate in forthcoming real-time studies. The further research will focus on evaluating the real-time performance of the proposed algorithm, applying it to develop a brain-controlled robotic arm for enhancing the rehabilitation of patients suffering from nerve injuries.

## 5 Conclusion

In this paper, a brain functional network feature extraction method based on DTF network features and graph theory features is proposed for classifying two MI tasks. The following conclusions were reached:

(1) Both DTF network features and graph theory features have demonstrated their effectiveness in classifying MI tasks and have positively contributed to the performance improvement of the CSP algorithm. Especially, the proposed CDGL incorporating DTF network features and graph theory features together achieves the highest classification accuracy among the other feature fusion methods. This indicates that analyzing the features of brain functional networks can provide essential information for distinguishing between different MI tasks.

(2) Increasing the number of channels provides more information about the EEG signals, which improves the model’s sensitivity and ability to discriminate between specific activities in brain regions. Specifically, as the number of channels increases, the ability to characterize the expression of EEG signals is enhanced.

(3) The Beta band functional brain network features exhibited superior performance enhancement for the CDGL algorithm compared to the Alpha band. This suggests that EEG signals in the Beta band may contain more valuable information and have a greater impact on the accuracy and robustness of the classification algorithm in MI tasks.

These findings contribute significantly to our understanding of the mechanisms underlying BCIs and MI tasks, offering fresh insights and novel possibility for further research in the field of neuroscience. Future studies can focus on exploring and refining feature extraction methods based on DTF and graph theory, extending their applicability to a wider range of tasks and practical applications.

## Data availability statement

The raw data supporting the conclusions of this article will be made available by the authors, without undue reservation.

## Ethics statement

The studies involving humans were approved by Ethics Review Committee of Inner Mongolia University of Technology. The studies were conducted in accordance with the local legislation and institutional requirements. The participants provided their written informed consent to participate in this study. Written informed consent was obtained from the individual(s) for the publication of any potentially identifiable images or data included in this article.

## Author contributions

PM: Formal analysis, Investigation, Methodology, Software, Writing – original draft. CD: Conceptualization, Funding acquisition, Resources, Supervision, Writing – review & editing. RL: Visualization, Writing – original draft. HZL: Data curation, Investigation, Writing – original draft. DL: Validation, Writing – original draft. XC: Software, Validation, Writing – review & editing. HL: Investigation, Software, Writing – review & editing.
